# Self-assembling soft structures for intracellular NO release and promotion of neurite outgrowth†
†Electronic supplementary information (ESI) available. See DOI: 10.1039/c6sc05017d



**DOI:** 10.1039/c6sc05017d

**Published:** 2017-06-20

**Authors:** Hilal Ahmad Pal, Saswat Mohapatra, Varsha Gupta, Surajit Ghosh, Sandeep Verma

**Affiliations:** a Department of Chemistry and Center for Environmental Science and Engineering , Indian Institute of Technology Kanpur , Kanpur 208016 , UP , India . Email: sverma@iitk.ac.in; b Department of Organic and Medicinal Chemistry , CSIR-Indian Institute of Chemical Biology Kolkata , 4, Raja S. C. Mullick Road , Jadavpur 700032 , WB , India

## Abstract

Nitric oxide (NO), an endogenously produced free radical species, is an extremely important signalling molecule in several biochemical processes related to neurotransmission, neuronal communication, and vasodilation, to name a few. Other than relying on endogenous synthesis, intracellular NO delivery presents an interesting challenge to fully exploit the therapeutic potential of this gaseous molecule. We have applied a self-assembling peptide conjugate strategy to devise a construct carrying a NO-release arm, which can be activated under standard redox conditions. Consequently, a tryptophan-based peptide carrier was designed, which self-assembled in the solution phase to afford soft nanospherical structures, and released NO in Neuro2a cell line, resulting in neurite outgrowth.

## Abstract 2

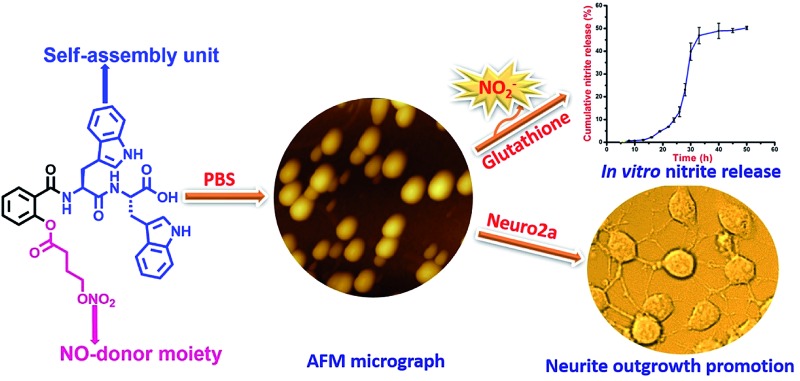
A tryptophan-based peptide conjugate with a NO-release arm was designed, which self-assembles in solution to afford soft spherical structures. This conjugate releases NO in a controlled fashion in Neuro2a cell line, resulting in neurite outgrowth.

## Introduction

Nitric oxide (NO) gas is a vital cellular signalling mediator, which regulates various biological functions within the cardiovascular, respiratory, and nervous systems.^1^ NO is biosynthesized inside cells through enzymatic conversion of l-arginine by many known (inducible) neuronal and endothelial isoforms of nitric oxide synthase (NOS).^2^ These are free radical species with high reactivity and often lead to the formation of other reactive nitrogen intermediates such as NO^–^, NO^+^, or sulphur nucleophile adducts such as *S*-nitrosothiols, which are redox-based, reversible NO-signalling pathways.^3^ Additional activities of NO include its crucial role in infection and innate immune response, and macrophage-mediated destruction of foreign pathogens.^4^

These pharmacological actions have catapulted interest in NO as a promising therapeutic agent.^5^ NO can act as a tumor promoter or regulate cell apoptosis *via* multiple mechanisms, including regulation of death receptor expression through nitrosylation of nucleophilic cysteine residues in caspase active sites, or by potentially inducing the expression of cytoprotective genes.^6^ Nitric oxide has been inextricably involved with induced neurite outgrowth and plays a crucial role in synaptogenesis, which is modulated by NO binding to soluble guanylate cyclase leading to its activation and production of cGMP.^7^ It was proposed that the release of NO followed by a clear understanding of its role in neuronal remodulation could lead to functional recovery of injured neurons.

The short half-life and chemical instability of NO under normal physiological conditions have hindered chemical strategies for the development of NO-based pharmaceuticals.^8^ It is clear that the therapeutic effects of NO-donors largely rely on the duration of release and the localized concentration achieved in this process. To address these issues, numerous NO donors, such as nitroprusside, *S*-nitrosothiols, *N*-diazeniumdiolates (NONOates), and organic nitrates, have been developed for the *in situ* release of NO.^9^ These agents are mostly low molecular weight compounds, which disperse rapidly to deliver low quantities of NO and are not easily manipulated for targeted delivery. Therefore, certain NO-releasing macromolecular scaffolds such as dendrimers, liposomes, nanoparticles, *etc.* have been employed to deliver NO in a controlled manner.^10^ Notably, the development of biocompatible nanoscale NO-delivery systems has also emerged as a promising strategy.^11^

Peptide-based nanoscale structures present exciting prospects in drug delivery, tissue engineering, and biosensing.^12^ These biocompatible soft structures offer encapsulation, are amenable to covalent modifications for targeted delivery, and present stimuli responsive characters. We and others have shown that the latter could be manifested by changes in pH, ionic strength, temperature, light, and redox environment to release bioactive molecules.^13,14^ Of these, photo-controlled mechanisms have served as versatile handles for NO release.^15^ For example, in a recent study, Kamei, Furukawa and co-workers developed two-photon laser activation of a NO-releasing coordination polymer, by embedding in a biocompatible PDS matrix, for the intracellular release of NO in HEK 293 cells.^16^

**Fig. 1:**
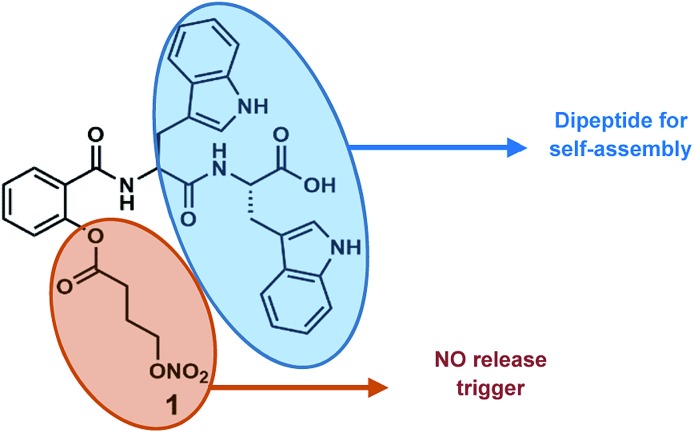
Molecular structure of conjugate **1**.

Given our interest in peptide-based soft structures, we decided to design a carrier system by combining the self-assembling character of aromatic dipeptides with a known NO-donor, 2-{[4-(nitrooxy)butanoyl]oxy} benzoic acid (nitrated aspirin).^17^ We envisioned that the presence of a tryptophan containing peptide will help assembly and a redox trigger will react with the NO donating handle for nitric oxide release. The NO-donor arm was selected due to its useful pharmacological actions such as anti-inflammatory activity, reduced gastro toxicity compared to aspirin, and platelet aggregation inhibition.^17^ Herein, we describe the design, synthesis, and *in vitro* nitrite and intracellular nitric oxide release from a novel NO releasing peptide conjugate, and the effect of nitric oxide on neurite growth in Neuro2a cells (Fig. 1).

## Results and discussion

**Fig. 2:**
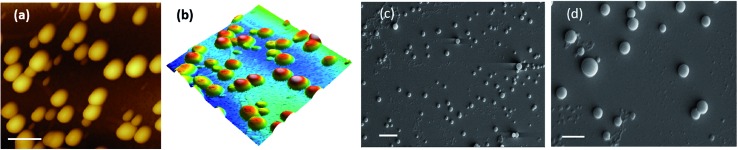
Microscopy analysis. (a) AFM micrograph of **1** (scale bar = 500 nm). (b) 3D height profile of the spherical structures of **1**. (c) SEM micrograph (scale bar = 2 μm). (d) Zoomed view (scale bar = 1 μm).

**Fig. 3:**
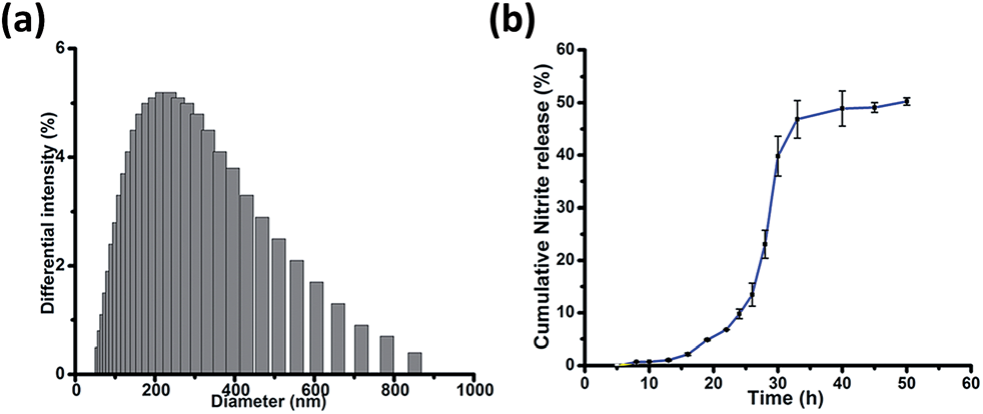
(a) Size distribution of **1** from the DLS measurements. (b) *In vitro* nitrite release from **1** (100 μM) on incubation with glutathione (5 mM) in PBS (pH 7.40) at 37 °C. The experiments were performed in triplicate (the points represent the average of three values).

Salicylic acid was conjugated to a ditryptophan peptide using solution phase protocols, followed by the attachment of the NO releasing arm and the target compound **1** was characterized for its stability and purity using analytical methods (Fig. S1 and S2 ESI†). Conjugate **1** afforded formation of spherical structures in an aqueous medium, as characterised using microscopy techniques (Fig. 2) such as atomic force microscopy (AFM) and scanning electron microscopy (SEM). As observed previously for a *C*_3_ symmetric Trp–Trp dipeptide, self-assembly of **1** afforded spherical structures ascribed to favourable hydrophobic π–π stacking and hydrogen bonding interactions,^18^ thus justifying our first design principle concerning the aspect of self-organization. Notably, the size distribution as assessed using dynamic light scattering measurements revealed an average hydrodynamic diameter of ∼300 nm for these spherical structures (Fig. 3a).

The pharmacological effects of organic nitrates are primarily mediated through their eventual transformation to nitric oxide. This activation can occur either *via* enzymatic or non-enzymatic processes. It has been suggested that endogenous thiols such as glutathione, cysteine, *N*-Ac-cysteine, *etc.* decompose organic nitrates to afford nitrite ions, while getting oxidized to the corresponding disulfides during this process.^19^ Cellular thiols facilitate the non-enzymatic conversion of organic nitrates for NO release and modulate nitrate-induced counter-regulatory mechanisms.^20^ For example, glutathione tripeptide affords the formation of *S*-nitrosoglutathione (GSNO), a rich bioavailable source of NO, which is regulated by GSNO reductase in an NADH-dependent reaction. The latter is known to be overexpressed in the hippocampal and cerebral brain regions and plays a crucial role in regulating NO release and its activity.^21^ In addition, thiol nitrosation at key protein targets could serve as a short-lived, post-translational regulatory mechanism to control neural plasticity and its involvement in the genesis of certain neurological diseases.^22^

Glutathione was used to study the nitrite release kinetics of **1** *via* incubation in phosphate buffer (5×, pH 7.40) at 37 °C. Following established protocols, the formation of nitrite was determined using Griess assay. This UV-Vis assay is based on the reaction between sulphanilamide, *N*-(1-naphthyl) ethylenediamine dihydrochloride, and nitrite in an acidic medium to afford azo dye formation, which can be quantitatively followed at 540 nm. Briefly, **1** (100 μM) in PBS (pH 7.4) was incubated with glutathione (GSH, 5 mM) at 37 °C. Aliquots (0.3 mL) of the mixture were taken at specified time points and treated with Griess reagent (0.1 mL), followed by re-incubation for 15 minutes at 37 °C. The absorption of the mixture was recorded at 540 nm on a UV spectrophotometer.^23^

The nitrite concentration of the mixture was calculated from the standard nitrite concentration–absorbance curve (Fig. S4, ESI†), which allowed the calculation of the percentage of nitrite released from **1**.

After a brief period of slow initial release, around 50% cumulative nitrite release was observed over a period of 50 h (Fig. 3b), which is in contrast to organic nitrates such as nitro-glycerine that release NO immediately upon incubation with glutathione.^24^ This difference in release kinetics could be attributed to the compact, self-assembled morphology of **1**. Nitrite release was not observed when **1** was incubated in phosphate buffer without glutathione, over a period of 50 h. **1** was further incubated with different concentrations of glutathione. An increase in glutathione concentration not only leads to faster nitrite release, but also to enhanced concentration of nitrite ions, as detected using the Griess assay (Fig. S5, ESI†).

We investigated the possible formation of *S*-nitrosoglutathione (GSNO) with the help of a time-dependent UV-Vis study (Fig. S6, ESI†). The results show the complete absence of the absorption characteristics of *S*-nitrosothiols (RSNO), which typically afford an intense absorption band between 330–350 nm and a weaker band in the 550–600 nm region.^25^ The absence of strong absorption at 330 nm indicated the lack of *S*-nitrosoglutathione formation arising from the incubation of **1** with glutathione, or that it remained undetected for our system.

The time-dependent degradation of **1** in the presence of glutathione was followed with the help of HPLC analysis. **1** (0.1 mM) was incubated with glutathione (5 mM) in 5× phosphate buffer solution (pH = 7.4), at 37 °C. Aliquots were drawn at various time intervals for HPLC analysis (monitored at 280 nm), where a peak corresponding to **1** (*R*_*t*_ = ∼12.65 min) decreased with respect to time and a new peak (*R*_*t*_ = ∼12 min) appeared (Fig. S7, ESI†), corresponding to the phenolic ester hydrolysis product (Fig. S13, ESI†). The release of nitrite ions was further confirmed with the help of ion exchange chromatography (Fig. S14, ESI†).

**Fig. 4:**
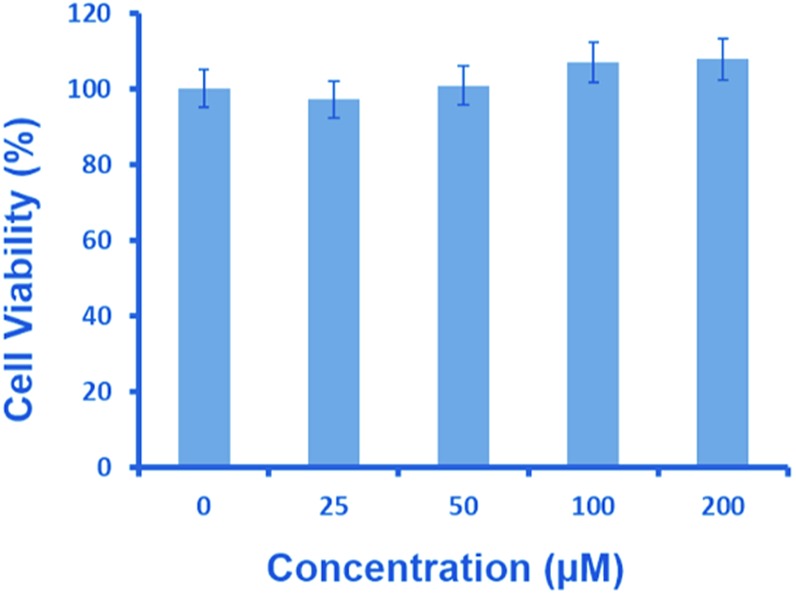
MTT assay in Neuro2a cells treated with **1** for 24 h.

Intracellular glutathione is widely distributed in cells and plays an important role in biochemical reactions. As Neuro2a cells contain a high amount of glutathione,^26^ it can be envisaged that a similar reaction can facilitate intracellular NO release from **1**. Thus, we decided to evaluate the potential of **1** in releasing nitric oxide in neuronal cells. As a start, MTT assay was performed to assess the toxicity of **1** in Neuro2a cells for several concentrations (25–200 μM) (Fig. 4).^27^ This revealed a lack of cytotoxicity, indicating the suitability of **1** for cell culture experiments.

**Fig. 5:**
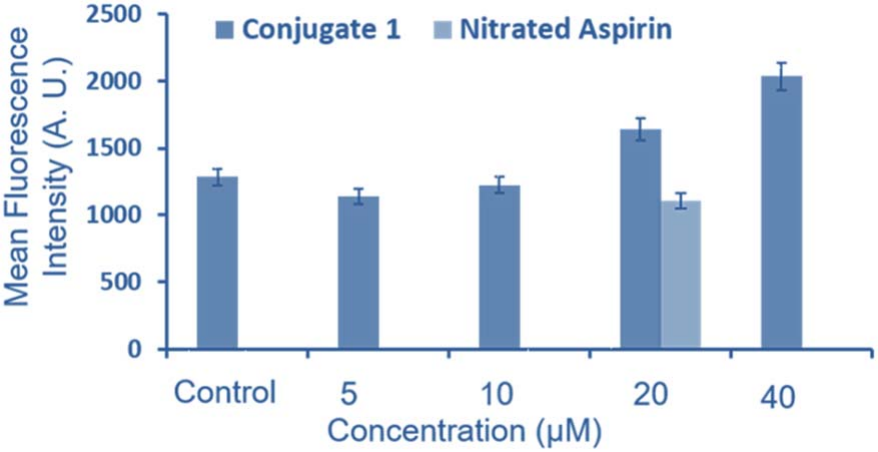
Results of the DAF-FM diacetate assay to assess intracellular release of NO after treatment with various concentrations of **1** in Neuro2a cells for 24 h. 20 μM of nitrated aspirin is used as a control to compare the potential of **1** for releasing intracellular NO.

NO release in the Neuro2a cell line was assessed after 24 h incubation, as reported for other NO donors.^28^ We checked the intracellular NO release *via* 4-amino-5-methylamino-2′,7′-difluorescein diacetate (DAF-FM diacetate) assay (Fig. 5).^29,30^ This assay revealed no change in the DAF fluorescence after treatment with 5 and 10 μm of **1** in the Neuro2a cells as compared to that of untreated cells (control). However, an appreciable increase in the fluorescence signal was observed after treatment with 20 and 40 μM of **1**, compared to the untreated cells.

To ascertain the beneficial role of **1**, we also compared the NO releasing potential of 2-{[4-(nitrooxy)butanoyl]oxy} benzoic acid (nitrated aspirin) as a control. The objective of this comparison was to demonstrate the purpose of conjugating the NO donor moiety (nitrated aspirin) with a ditryptophan peptide, which affords the formation of aggregated spherical structures. Interestingly, we observed that the NO-releasing ability of nitrated aspirin at 20 μM is significantly lower compared to that of **1** at the same concentration. These results clearly indicate that **1** has significant potential of releasing NO in cellular environments in the aggregated form.

**Fig. 6:**
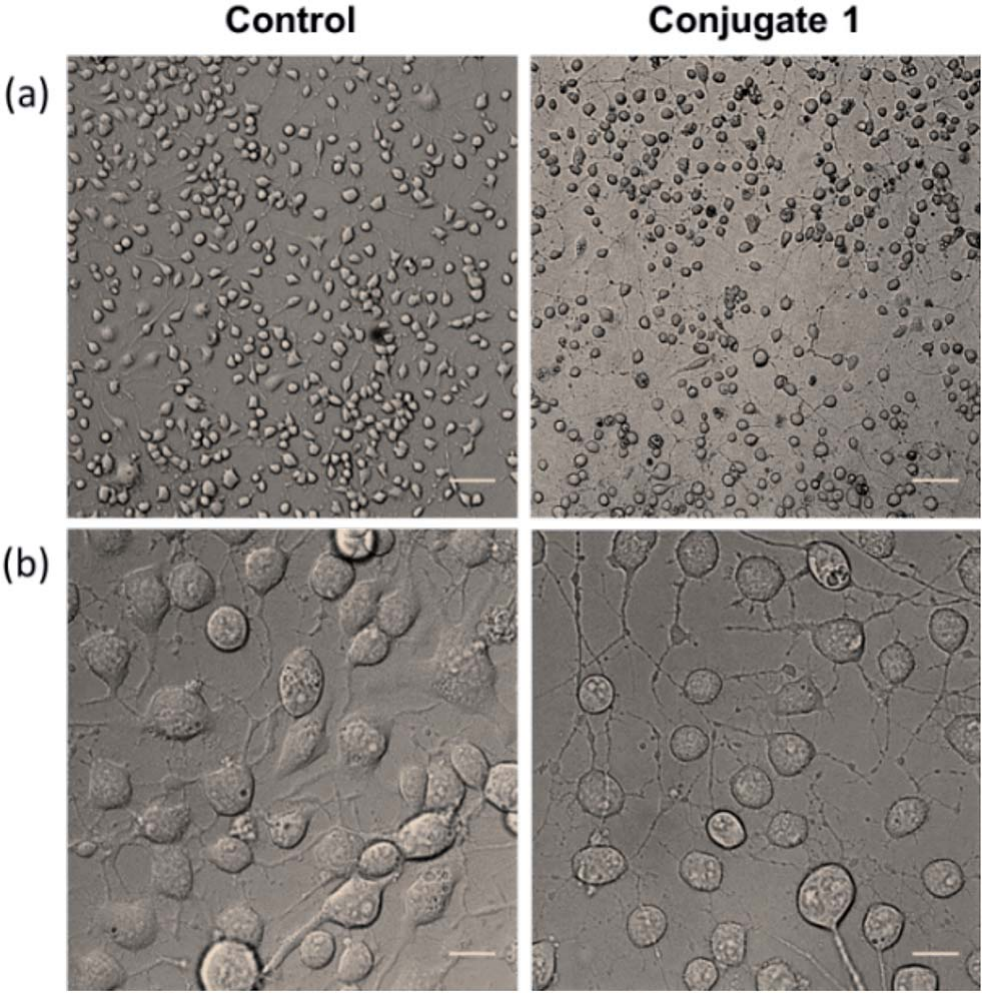
DIC images representing the promotion of neurite outgrowth in Neuro2a cells shown in lower magnification (a) where scale bars correspond to 100 μm and higher magnification (b) where scale bars correspond to 20 μm.

**Fig. 7:**
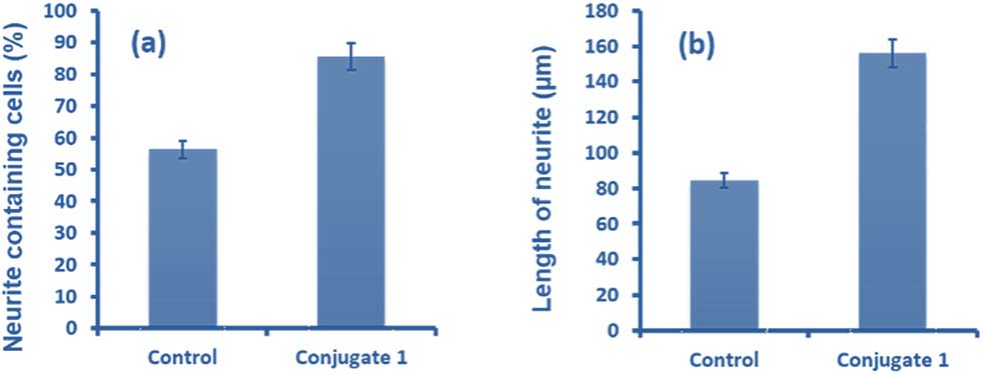
Quantitative analysis showing (a) the percentage of neurite containing cells and (b) the length of neurites after treatment with **1**, compared to untreated cells.

Neurite outgrowth of neuron cells indicates that neuron cells are healthy and active. We checked the potential of **1** in neurite outgrowth using phase contrast or differential interference contrast (DIC) microscopy. Numerous reports have described the use of this method to discriminate between neurite bearing and non-bearing cells, as it is an excellent method to gain contrast in transparent specimens.^31^ In a typical experiment, Neuro2a cells were treated with **1** (20 μM) followed by observation in DIC mode. The images clearly demonstrated significant neurite outgrowth in cells treated with **1**, compared to untreated (control) cells (Fig. 6a and b). The number of cells showing neurite outgrowth, as well as neurite length, was quantified using cellSens software, which clearly showed ∼30% enhancement in the number of neurite-bearing cells (Fig. 7a), while the neurite length almost doubled compared to that of the control cells (Fig. 7b). These results further confirm the potential of **1** for promoting neurite outgrowth in healthy neuronal cells.

Taken together, these results confirm the premise of combining a self-assembling motif appended with organic nitrate as a nitric oxide release trigger, which requires thiol for decomposition. We observed not only formation of homogeneously sized soft structures in solution from **1**, but also the *in vitro* release of nitrite ions in the presence of glutathione. Notably, this strategy was also validated in a neuronal cell line where the release of nitric oxide and new neurite outgrowth was detected. These studies open new possibilities of extending the suggested design with target-specific soft structures, investigations concerning biological outcomes, and optimizing such vehicles for releasing other gasotransmitters.

## Conclusions

In conclusion, we have described a NO releasing novel peptide conjugate platform, combining the virtues of a self-assembling system with a nitric oxide precursor trigger, which reacts under redox conditions to release NO *in vitro* as well as within neuronal cells. This approach offers immense promise in discovering better biocompatible handles to study the physiology of sustained NO release under controlled conditions for topical and systemic applications such as wound healing, erectile dysfunction, and a host of vascular inflammatory conditions.
