# Supplementary material for: Self-assembling soft structures for intracellular NO release and promotion of neurite outgrowth
Source: Chem Sci. 2017 Jun 20;8(9):6171–5. doi: 10.1039/c6sc05017d (PMC5627600; doi:10.1039/c6sc05017d)
Supplement: Supplementary file 1 [file SC-008-C6SC05017D-s001.pdf]

Electronic Supplementary Material (ESI) for Chemical Science.
This journal is © The Royal Society of Chemistry 2017

Self-assembling soft structures for intracellular NO release and promotion of neurite outgrowth

Hilal Ahmad Pal, [a] Saswat Mohapatra, [b] Varsha Gupta, [b] Surajit Ghosh [b] and Sandeep Verma*[a]

[a]Department of Chemistry and Center for Nanoscience and Soft Nanotechnology, Indian Institute of Technology Kanpur, Kanpur-208016 (UP), India.

Fax: (+) 91-512-2597436, E-mail: sverma@iitk.ac.in

[b]Department of Organic and Medicinal Chemistry, CSIR-Indian Institute of Chemical Biology Kolkata 4, Raja S. C. Mullick Road, Jadavpur-700032 (WB), India.

Contents

Materials and methods
Microscopy studies-AFM and SEM
High-performance liquid chromatography (HPLC)
Scheme 1 Synthetic route of **1**
Synthesis and characterization of **1**
Fig. S1 Analytical HPLC spectrum of **1**
Fig. S2 Stability of **1** in PBS aftter 0 h, 6 h and 12 h of incubation
In vitro nitrite release study
Fig. S3 Representative UV-Vis spectrum after incubation of **1** with glutathione in PBS
Fig. S4 Standard nitrite (NO_2^-) concentration-absorption curve.
Fig. S5 Nitrite release from **1** as a function of glutathione concentration
Fig. S6 Time dependent UV Vis spectrum of **1** incubated with glutathione
Fig. S7 Time dependent HPLC spectrum of **1** incubated with glutathione in phosphate buffer solution 37 °C.
Cell culture studies
Fig. S8 Zoomed images of neurite outgrowth in neuron cells.
Fig S9 IR spectrum of 1
Fig. S10 ^{1}H NMR spectrum of **1**
Fig. S11 ^{13}C NMR spectrum of **1**
Fig. S12 HRMS-ESI spectrum of **1**
Fig. S13 HRMS-ESI spectrum of **1** after nitrite release

Materials and methods

Acetonitrile (CH_3CN), dichloromethane (DCM), methanol (MeOH) and triethylamine (TEA) were distilled following standard procedures prior to use. Benzyl chloroformate, 4-(dimethylamino) pyridine and *tert*-butyl bromide were obtained from Spectrochem (Mumbai, India). L-tryptophan, benzyltriethylammonium chloride, 1-hydroxybenzotriazole, trifluoroacetic acid, *N, N*-Diisopropylethylamine were purchased from Avra synthesis Pvt. Ltd. (Hyderabad, India); 10% Pd/C and 4-bromobutyric acid were obtained from Alfa Aesar; *N*-(3-dimethylaminopropyl)-*N*'-ethylcarbodiimide hydrochloride and sodium bicarbonate were obtained from Sisco Research laboratories Pvt. Ltd. (Mumbai India); silver nitrate and sodium carbonate were purchased from Rankem; salicylic acid was obtained from Merck; potassium carbonate, hydrochloric acid and DAF-FM Diacetate (4-Amino-5-Methylamino-2', 7'-Difluorofluorescein Diacetate) were purchased from Thermo Fisher Scientific; Triethyl amine was procured from S D Fine Chem Limited (Mumbai) and were used without further purification. 5-diphenyltetrazolium bromide (MTT) and dulbecco's modified eagle's medium (DMEM) were purchased from Sigma Aldrich. 4-(2-hydroxyethyl)-1-piperazineethanesulfonic acid (HEPES) was purchased from Himedia and $NaHCO_3$ (Sodium bicarbonate) was obtained from Merck. Kanamycin sulfate, penicillin-streptomycin and fetal bovine serum were purchased from Gibco. Neuro2a cell line was purchased from National Centre for Cell Science (NCCS) Pune, India. ^1H and ^{13}C NMR spectra were recorded on JEOL-JNM ECS 400 model operating at 400 and 100 MHz, respectively. HRMS spectra were recorded at IIT Kanpur, India, on Waters, Q-Tof Premier Micromass HAB 213 mass spectrometer using capillary voltage 2.6-3.2 Kv. UV-Vis absorption spectra were recorded on Jasco V-670 spectrophotometer with 10 mm quartz cell at 25 °C. Dynamic light scattering for Particle size distribution was done using Delsa Nano from Beckman Coulter India. Griess reagent was prepared in laboratory according to reported literature procedure.[1]

Microscopy studies:

Atomic Force Microscopy (AFM): 10 µL aliquot of freshly prepared solution of **1** (100 µM in water) was deposited on clean glass surface and spread uniformly on it. The sample was dried in air at room temperature for overnight and was subsequently scanned with an atomic force

microscope (Agilent Technologies 5500 AFM/SPM) at room temperature. Scanning was carried out under acoustic AC mode (AAC) with a force constant of 0.6 N/m. The sample was mounted on the XY stage of the AFM and the integral video camera (NAVITAR, Model N9451A-USO6310233 with the Fiber-light source, MI-150 high intensity illuminator from Dolan-Jenner Industries) was used to locate the regions of the interest. Silicon nitride cantilever with a resonant frequency of 170 kHz was used. The scanner model N9520AUSO7480152.xml was calibrated and used for imaging. Images were taken in air at room temperature with a scan speed of 1.0 lines/s. Data acquisition and analysis was done using Pico View® 1.8 and Pico Image® Basic software respectively.

Scanning Electron Microscopy (SEM): Field emission scanning electron microscopy (FESEM) images were acquired on FEI QUANTA 200 microscope, equipped with a tungsten filament gun, operating at working distance 4.0 mm and 7.99 kV. A 10 µL aliquot of freshly prepared solution of **1** (100 µM in water) was placed on glass cover slip and was allowed to dry at room temperature for overnight followed by drying under high vacuum for another 30 minutes. The sample was gold coated for 45 s and then imaged with FESEM.

High-performance liquid chromatography (HPLC): HPLC analyses were performed with a HPLC system (Agilent technologies 1260 infinity) equipped with a quaternary pump (G1311B), auto liquid sampler (G1329B), Diode array detector (G1315D) and analytical scale-fraction collector (G1364C). Instrument control, data acquisition and data analysis was performed using a ChemStation software (Agilent Technologies, Workingham, UK). A ZORBAX Eclipse plus C 18 (250 x 4.6 mm) column with 5 µm particle size at room temperature was used. Mobile phase consisted of methanol/ water (10:90), and the flow rate was 1.0 mL/min. Injection volume was 25 μL and the column effluent was monitored at 280 nm.

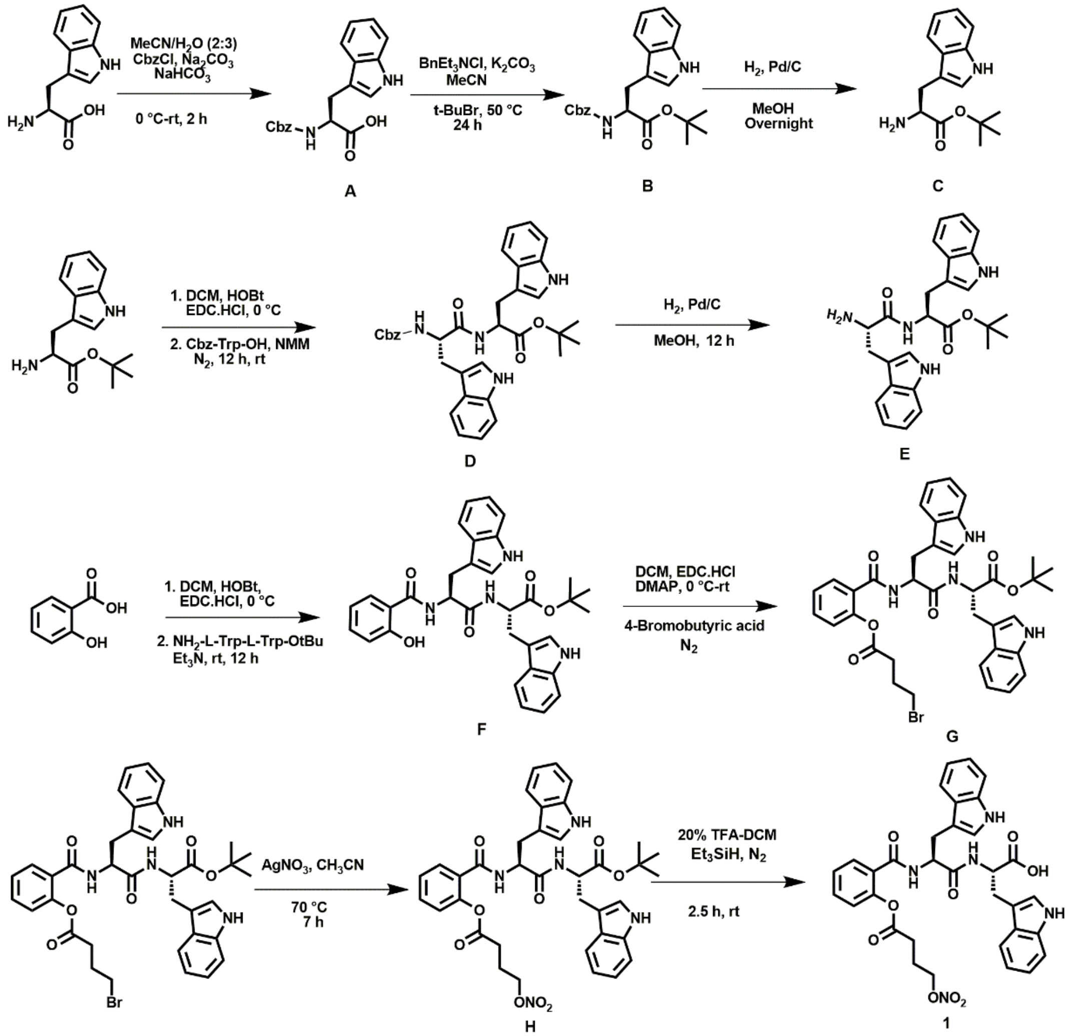

Scheme 1. Synthetic route of **1.**

Synthesis and characterization of 1:

Synthesis of *N*-(benzyloxycarbonyl)-L-tryptophan (A): To a clear solution of L-tryptophan (3 g, 14.68 mmol) in 2:3 mixture of water/acetonitrile was added $NaHCO_3$ (1.85 g, 22.03 mmol) and Na_2CO_3 (2.33 g, 22.03 mmol). The resulting turbid solution was cooled to 0 °C in ice bath. Benzyloxycarbonyl chloride (2.096 mL, 14.68 mmol) was added slowly to this mixture and the solution was stirred for two hours at room temperature after which no starting material was left. The solution was acidified by dropwise addition of aqueous 1 N HCl solution and acetonitrile was removed by rotary evaporation. The mixture was transferred to the separating funnel and was extracted thrice with ethyl acetate. The combined organic layer was washed with brine and dried over sodium sulphate. Concentration of the organic phase under reduced pressure gave *N*-Cbz-L-tryptophan as white solid. Yield (4.47 g) 90%. R_f : 0.7 (4:1 CH_2Cl_2/MeOH); ESI-HRMS: $[M+Na]^+$, calculated = 361.1164, found = 361.1169; ^1H NMR (400 MHz, DMSO-D_6, 25 °C, TMS) δ(ppm): 2.92-2.98 (m, 1H), 3.11-3.16, (m,1H), 4.16-4.22 (m,1H), 4.89-4.97 (m, 2H), 6.92-6.96 (t, 1H, J = 6.88 Hz, J = 8.24 Hz), 7.01-7.04 (t, 1H, J = 8. 24 Hz, J = 6.88 Hz), 7.09-7.14 (s, Broad, 1H,), 7.23-7.31 (m, 6H), 7.49-7.55 (m, 2H), 10.87 (s, 1H). ^{13}C NMR (100 MHz, DMSO-D_6, 25 °C, TMS) δ (ppm): 26.94, 54.79, 65.81, 110.33, 111.93, 118.64, 118.79, 121.59, 124.57, 127.63, 128.26, 128.65, 136.63, 137.50, 156.52, 174.24

Synthesis of *N*-(benzyloxycarbonyl)-L-tryptophan-*tert*-butyl ester (B): *N*-(benzyloxycarbonyl)-L-tryptophan (2.5 g, 7.38 mmol), benzyltriethylammonium chloride (1.68 g, 7.38 mmol) and K_2CO_3 (6.63, 48.02 mmol) mixture in acetonitrile (70 mL) was stirred vigorously for five hours. Tertiary butyl bromide (8.72 mL, 77.72 mmol) was added slowly to this mixture and it was heated at 50 °C for three hours. The heating was turned off and the reaction mixture was left for stirring for 24 hours at room temperature. After this reaction mixture was filtered and acetonitrile was evaporated under reduced pressure. The residue was dissolved in water and ethyl acetate mixture. The aqueous solution was extracted three times with ethyl acetate and the combined organic layer was washed with brine and dried over anhydrous sodium sulphate. The crude compound was purified by silica gel column chromatography us 10% ethyl acetate/hexane

solvent system to give pure title compound. Yield 2 g (70%). R_f : 0.8 (3% methanol in dichloromethane); ESI-HRMS: $[M+H]^+$, calculated = 395.1971, found = 395.1970; ^1H NMR (400 MHz, DMSO-D_6, 25 °C, TMS) δ (ppm): 1.26 (s, 9H), 2.90-2.96 (m,1H), 3.04-3.09 (m, 1H), 4.12-4.17 (m,1H), 4.93-4.95 (m, 2H), 6.92-6.96 (t, 1H, J = 7.2 Hz), 7.01-7.04 (t, 1H, J = 7.2 Hz), 7.09-7.14 (S, 1H), 7.20-7.35 (m, 6H), 7.46-7.48 (d, 1H, J = 7.6 Hz, J= 7.6 Hz), 7.60-7.62 (d, 1H, J = 8 Hz), 10.80 (s, 1H). ^{13}C NMR (100 MHz, DMSO-D_6, 25 °C, TMS) δ (ppm): 26.94, 28.0, 54.79, 65.81, 80.0, 110.33, 111.93, 118.64, 118.79, 121.59, 124.57, 127.63, 128.26, 128.65, 136.63, 137.50, 156.52, 174.24

Synthesis of L-tryptophan-*tert*-butyl ester (C): To a stirred solution of *N*-(benzyloxycarbonyl)-L-tryptophan-*tert*-butyl ester (1.8 g, 4.56 mmol) in methanol (20 mL) was added catalytic amount of 10% Pd/C (0.2 g).The mixture was stirred under hydrogen atmosphere at room temperature for overnight after which no starting material could be detected by TLC analysis. The presence of amine group was confirmed by developing it on TLC and staining by ninhydrin. The catalyst was removed by filtration through a pad of celite and washed with methanol several times. The filtrate was concentrated under reduced pressure to give C in quantitative yield. This compound was used in next step without any further purification. R_f : 0.3 (3% methanol in dichloromethane); ESI-HRMS: $[M+H]^+$, calculated = 261.1603, found = 261.1601; ^1H NMR (500 MHz, DMSO-D_6, 25 °C, TMS) δ (ppm): 1.24 (s, 9H), 2.80-2.85 (m, 1H), 2.91-2.96 (m, 1H), 3.45-3.48 (t, 1H, J = 6.85 Hz, 6.9 Hz), 6.91-6.95 (t, 1H, J = 7.2 Hz), 6.99-7.03 (t, 1H, J = 6.8, 7.6 Hz), 7.07 (s, 1H), 7.26-7.29 (m, 1H), 7.47-7.49 (d, 1H, J = 7.6 Hz), 10.75-10.78 (br, 1H); ^{13}C NMR (100 MHz, DMSO-D_6, 25 °C, TMS) δ (ppm): 28.12, 31.49, 56.26, 80.10, 110.78, 111.80, 118.70, 119.01, 121.34, 124.07, 128.02, 136.59, 175.10

Synthesis of *N*-(benzyloxycarbonyl)-L-tryptophan-tryptophan-*tert*-butyl ester (D): *N*-(benzyloxycarbonyl)-L-tryptophan (1.29 g, 3.84 mmol) and 1-hydroxybenzotriazole (0.622 g, 4.60 mmol) were dissolved in dichloromethane (25 mL) and *N, N*-Dimethylformamide (2 mL) mixture under nitrogen atmosphere and subsequently cooled to 0 °C in ice bath. 1-ethyl-3-(3-dimethylaminopropyl) carbodiimide hydrochloride was added at 0 °C and the reaction mixture was stirred for one hour. Next L-tryptophan-*tert*-butyl ester (1 g, 3.84 mmol) was added followed

by addition of *N*-methyl morpholine (0.84 mL, 7.68 mmol) and the stirring was continued for 12 hours at room temperature. After the completion of the reaction, the reaction mixture was washed twice with 10% aqueous $NaHCO_3$ solution and 1 N aqueous HCl solution respectively. The combined organic layer was washed with brine and dried over anhydrous sodium sulphate. The crude compound was purified through silica gel column chromatography using 30% (v/v) ethyl acetate/hexane as eluent to give pure *N*-(benzyloxycarbonyl)-L-tryptophan-tryptophan-*tert*-butyl ester as white powder. Yield 1.67 g (75%); R_f : 0.4 (3% methanol in dichloromethane); ESI-HRMS: $[M+H]^+$, calculated = 581.2764, found = 581.2761; ^{1}H NMR (400 MHz, DMSO-D_6, 25 °C, TMS) δ (ppm): 1.24 (s, 9H), 2.83-2.89 (m, 1H), 3.01-3.14 (m, 3H), 4.29-4.35 (m, 1H), 4.40-4.57 (m, 1H), 4.83-4.94 (m, 2H), 6.91-6.97 (m, 2H), 7.00-7.05 (m, 2H), 7.09-7.14 (m, 2H), 7.18-7.20 (d, 2H, J = 6.4 Hz), 7.23-7.31 (m, 6H), 7.49-7.51 (d, 1H, J = 7.8 Hz), 7.60-7.62 (d, 1H, 7.8 Hz), 8.32-8.34 (1H, d, 7.36 Hz), 10.76 (s, 1H), 10.83 (s, 1H). ^{13}C NMR (100 MHz, DMSO-D_6, 25 °C, TMS) δ (ppm): 27.45, 27.88, 28.39, 54.18, 55.77, 65.76, 80.95, 109.96, 110.61, 112.0, 118.70, 118.76, 119.14, 121.49, 124.24, 124.47, 127.75, 127.99, 128.19, 128.81, 136.60, 137.50, 156.31, 171.35, 172.44

Synthesis of L-tryptophan-tryptophan-*tert*-butyl ester (E): *N*-(benzyloxycarbonyl)-L-tryptophan-tryptophan-*tert*-butyl ester (1.5 g, 2.58 mmol) was dissolved in methanol (15 mL) followed by addition of catalytic amount of 10% Pd/C (0.2 g).The mixture was stirred under hydrogen atmosphere for 12 hours at room temperature. The catalyst was removed by filtration through a pad of celite and was washed with methanol several times. The filtrate was evaporated under reduced pressure to afford E as white solid which was used in next step without further purification. Yield 1.0 g (92%), R_f : 0.2 (3% methanol in dichloromethane); ESI-HRMS: $[M+H]^+$, calculated = 447.2396, found = 447.2393; ^{1}H NMR (400 MHz, DMSO-D_6, 25 °C, TMS) δ (ppm): 1.26 (s, 9H), 2.92-3.04 (m, 4H), 3.42-3.45 (m, 1H), 4.47-4.52 (m, 1H), 6.82-6.85 (t, 1H, J = 8 Hz, 7.2 Hz), 6.92-6.95 (t, 1H, J = 7.6 Hz, 6.8 Hz), 6.97-7.03 (m, 3H), 7.12 (s, 1H), 7.26-7.30 (t, 3H, J = 8.4 Hz, 7.6 Hz), 7.51-7.53 (d, 1H, J = 7.6 Hz), 8.07-8.09 (d, 1H, J = 8.4 Hz), 10.77 (s, 1H), 10.83 (s, 1H); ^{13}C NMR (100 MHz, DMSO-D_6, 25 °C, TMS) δ (ppm): 27.54, 31.05, 53.63, 55.40, 81.18, 109.49, 110.89, 111.89, 118.38, 121.45, 124.04, 124.47, 127.81, 128.02, 136.53, 136.91, 171.40, 175.05

Synthesis of (2-Hydroxybenzoyl)-L-tryptophan-tryptophan-*tert*-butyl ester (F): To a mixture of salicylic acid (0.402 g, 2.91 mmol) and 1-hydroxybenzotriazole (0.534 g, 3.49 mmol) in dichloromethane (10 mL) stirred under nitrogen atmosphere was added 1-ethyl-3-(3-dimethylaminopropyl) carbodiimide hydrochloride (0.669 g, 3.49 mmol) at 0 °C. After half an hour, L-tryptophan-tryptophan-*tert*-butyl ester (1.30 g, 2.91 mmol) was added which was followed by addition of triethylamine (0.81 mL, 5.82 mmol). The reaction mixture was left for stirring for 12 hours at room temperature after which no starting material was left. The mixture was washed thrice successively with 10% aqueous $NaHCO_3$ solution and 1 N aqueous HCl solution. The combined organic layer was dried over anhydrous sodium sulphate and evaporated under reduced pressure. The crude compound was purified through silica gel column chromatography (ethyl acetate/hexane, 25% v/v). Yield 1.31 g (80 %); R_f : 0.7 (3% methanol in dichloromethane); ESI-HRMS: $[M+H]^+$, calculated = 567.2607, found = 567.2605; ^1H NMR (400 MHz, DMSO-D_6, 25 °C, TMS) δ (ppm): 1.25 (s, 9H), 3.03-3.16 (m, 3H), 3.20-3.24 (m, 1H), 4.43-4.48 (m, 1H), 4.80-4.85 (m, 1H), 6.80-6.84 (t, 2H, J = 6.8 Hz, 9.2 Hz), 6.89-6.95 (m, 2H), 6.98-7.04 (m, 2H), 7.12-7.16 (m, 2H), 7.25-7.33 (m, 3H), 7.49-7.51 (d, 1H, J = 8 Hz), 7.64-7.66 (d, 1H, J = 8 Hz), 7.85-7.87 (m, 1H), 8.58-8.60 (d, 1H, J = 7.2 Hz), 8.79-8.81 (d, 1H, J = 8.4 Hz), 10.74 (s,1H), 10.83 (s, 1H), 11.94 (s, 1H); ^{13}C NMR (100 MHz, DMSO-D_6, 25 °C, TMS) δ (ppm): 27.56, 27.67, 28.48, 53.71, 54.50, 81.35, 109.79, 110.40, 112.17, 116.14, 117.55, 119.0, 119.33, 121.53, 123.93, 124.38, 127.73, 129.18, 134.05, 136.78, 159.28, 167.85, 171.17, 172.31

Synthesis of 2-(4-bromobutanoyloxy) benzoyl-L-tryptophan-tryptophan-*tert*-butyl ester (G): 1-ethyl-3-(3-dimethylaminopropyl) carbodiimide hydrochloride (0.446 g, 2.32 mmol) was added to a solution of 4-bromobutyric acid (0.324 g, 1.94 mmol) in dichloromethane (10 mL) at 0 °C under nitrogen atmosphere, followed by addition of 4-dimethyl aminopyridine (0.0237 g, 0.194 mmol).

Next after half an hour, (2-Hydroxybenzoyl)-L-tryptophan-tryptophan-*tert*-butyl ester (1.10 g, 1.94 mmol) was added and the stirring was continued for six hours at room temperature. After the reaction was over, the reaction mixture was washed with aqueous 1 N HCl solution twice and the combined organic phase was dried over anhydrous sodium sulphate. The crude compound was purified through silica gel column chromatography by using hexane and ethyl

acetate (75:25) solvent system to give G as white solid. Yield: 1.18 g (85%). R_f : 0.6 (3% methanol in dichloromethane); ESI-HRMS: $[M+NH_4]^+$, calculated = 732.2397, found = 732.2403; ^{1}H NMR (400 MHz, DMSO-D_6, 25 °C, TMS) δ (ppm): 1.24 (s, 9H), 1.84-1.92 (m, 2H), 2.25-2.43 (m, 2H), 3.01-3.21 (m, 4H), 3.33-3.37 (m, 2H), 4.44-4.49 (m, 1H), 4.74-4.78 (m, 1H), 6.89-6.96 (m, 2H), 6.99-7.04 (m, 2H), 7.09-7.14 (m, 3H), 7.24-7.31 (m, 3H), 7.43-7.47 (m, 2H), 7.49-7.51 (d, 1H, J = 7.6 Hz), 7.56-7.58 (d, 1H, J = 8 Hz), 8.14-8.16 (d, 1H, 7.6 Hz), 8.41-8.43 (d, 1H, J = 7.2 Hz), 10.79 (s, 1H), 10.83 (s, 1H). ^{13}C NMR (100 MHz, DMSO-D_6, 25 °C, TMS) δ (ppm): 27.57, 27.85, 28.34, 32.56, 34.14, 53.77, 54.20, 80.70, 109.82, 110.41, 111.94, 118.85, 121.38, 121.48, 123.93, 124.18, 126.30, 127.73, 128.91, 131.95, 136.61, 148.34, 165.02, 171.04, 171.42, 171.96

Synthesis of 2-{[4-(Nitrooxy)butanoyl]oxy} benzoyl-L-tryptophan-tryptophan-*tert*-butyl ester (H): Compound G (1 g, 1.39 mmol) was dissolved in acetonitrile followed by the addition of $AgNO_3$ (0.593 g, 3.49 mmol) and was subsequently heated at 70 °C for 7 h. After the completion of the reaction, the mixture was filtered through celite and the solvent was concentrated under reduced pressure. The residue was dissolved in ethyl acetate (20 mL) and water (20 mL) mixture and extracted twice with ethyl acetate. The combined organic layer was dried, filtered, and concentrated under reduced pressure. The crude product thus obtained was purified by silica gel column chromatography [75:25 PE/EtOAc v/v] to give pure title compound. Yield 0.827 g (85%). ESI-HRMS: $[M+H]^+$, calculated = 698.2826, found = 698.2823. R_f : 0.7 (3% methanol in dichloromethane); ^{1}H NMR (400 MHz, DMSO-D_6, 25 °C, TMS) δ (ppm): 1.24 (s, 9H), 1.72-1.76 (m, 2H), 2.22-2.28 (m, 1H), 2.32-2.40 (m, 1H), 3.02-3.21 (m, 4H), 4.28-4.34 (t, 2H, J = 6.4 Hz), 4.44-4.50 (m, 1H), 4.74-4.79 (m, 1H), 6.88-7.04 (m, 4H), 7.10-7.14 (m, 3H), 7.24-7.30 (m, 3H), 7.44-7.55 (m, 4H), 8.11-8.13 (d, 1H, J = 8.24 Hz), 8.39-8.41 (d, 1H, J = 7.32 Hz), 10.79 (s, 1H), 10.82 (s 1H). ^{13}C NMR (100 MHz, DMSO-D_6, 25 °C, TMS) δ (ppm): 21.03, 28.02, 53.85, 54.45, 73.34, 80.88, 110.20, 110.42, 111.88, 111.87, 118.96, 121.30, 124.31, 126.34, 127.75, 128.88, 129.54, 131.97, 136.58, 148.28, 165.17, 171.12, 171.56, 172.0

Synthesis of 2-{[4-(Nitrooxy)butanoyl]oxy} benzoyl-L-tryptophan-tryptophan-OH (1): Compound H (0.5 g, 0.71 mmol) was dissolved in dichloromethane (20 mL). To this triethyl silane (0.347 mL, 2.14 mmol) and triflouro acetic acid (5 mL) under nitrogen atmosphere at room temperature were added. The reaction was complete within 2.5 h. The solvent was removed in vacuo and the residue washed with diethyl ether. The crude product was purified by column chromatography (5% MeOH/DCM). Yield 0.3 g (66%). R_f : 0.3 (10% methanol in dichloromethane); M.P = 145-147 C°; $[\alpha]_D^{25}$ = -10.81 (c 0.148, methanol); ESI-HRMS: $[M+H]^+$, calculated = 642.2200, found = 642.2203; ^{1}H NMR (400 MHz, DMSO-D_6, 25 °C, TMS) δ (ppm): 1.67-1.80 (m, 2H), 2.16-2.29 (m, 1H), 2.29-2.40 (m, 1H), 2.99-3.10 (m, 2H), 3.16-3.21 (m, 2H), 4.29-4.33 (t, 2H, J = 6.8 Hz, J = 6.4 Hz), 4.50-4.56 (m, 1H), 4.71-4.77 (m, 1H), 6.87-6.95 (m, 2H), 6.97-7.03 (m, 2H), 7.08-7.15 (m, 3H), 7.23-7.30 (m, 3H), 7.43-7.55 (m, 4H), 8.11-8.13 (d, 1H, J = 8.4 Hz), 8.27-8.29 (d, 1H, J = 7.2 Hz), 10.77 (s, 1H), 10.83 (s, 1H). ^{13}C NMR (100 MHz, DMSO-D_6, 25 °C, TMS) δ (ppm): 21.76, 27.67, 29.86, 53.54, 54.02, 73.10, 110.13, 110.43, 111.74, 111.86, 118.70, 118.88, 121.43, 123.87, 124.14, 126.32, 127.79, 127.94, 128.92, 129.81, 131.94, 136.55, 148.29, 156.13, 165.13, 171.10, 171.77, 173.72.

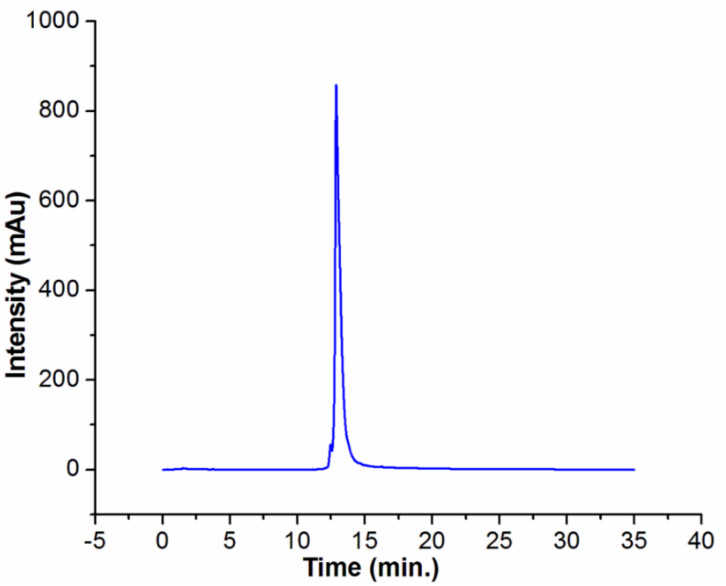

Fig. S1 Ananlytical HPLC spectrum of **1**

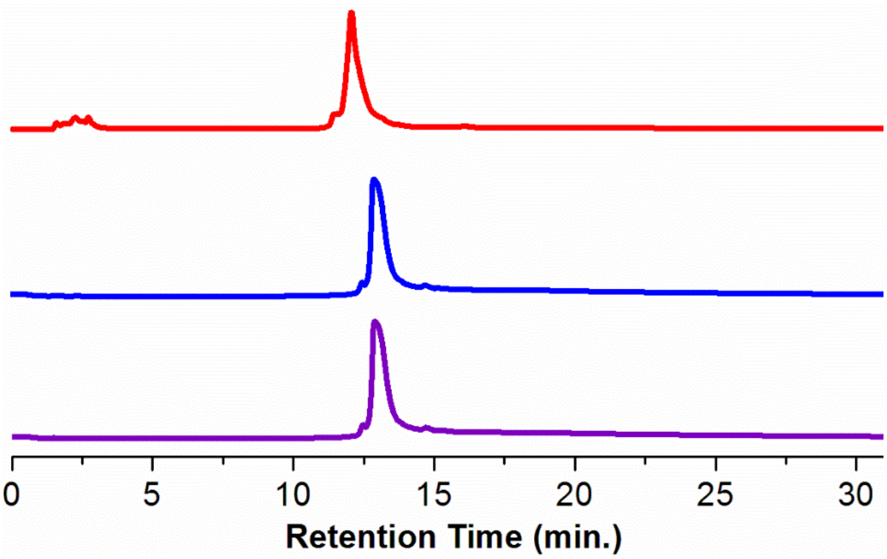

Fig. S2 Stability of **1** in PBS after 0 h, 6 h and 12 h incubation at 37 °C.

***In vitro* nitrite release study:** *In vitro* nitrite release was studied by incubating **1** (6 mL of 100 µM) with glutathione (5 mM) in phosphate buffer solution (pH 7.40) at 37 °C. Nitrite ions released were measured indirectly using Griess assay. Phosphate buffer solution (PBS) was prepared by dissolving KH_2PO_4 (50 mM) and Na_2HPO_4 (50 mM) in distilled water. The solution was titrated to physiological pH (7.40) using 0.01 N NaOH. Griess reagent was prepared by dissolving sulfanilamide (0.232 M) and *N*-(1-Naphthyl)ethylenediamine dihydrochloride (0.0077 M) in a mixture of 85% H_3PO_4 (10 mL) and distilled water (90 mL). PBS was used as blank control solution. It was incubated at 37 °C for one hour and later treated with Griess reagent followed by re-incubation of the mixture at 37 °C for 15 minutes. Sodium nitrite solutions with concentrations between 1-100 µM were prepared by diluting the stock solution of sodium nitrite (2 mM) with phosphate buffer solution followed by incubation at 37 °C. Griess reagent was then added to each of these nitrite solutions and the mixture was re-incubated at 37 °C for 15 minutes in each case. These solutions of different nitrite concentrations were used to plot a standard concentration–absorbance curve, which allowed the calculation of nitrite released from **1**.[1]

Stock solution of **1** (2 mM) was prepared in phosphate buffer solution. An aliquot (250 µL) of this solution was diluted with PBS followed by addition of glutathione so that the final concentrations of **1** and glutathione in the solution are 0.1 mM and 5 mM respectively. The

mixture was incubated at 37 °C. At specified time points, 300 µL of the mixture were withdrawn and treated with 100 µL Griess reagent followed by re-incubation at 37 °C for 15 minutes. The ultraviolet absorbance values for the blank control solution, conjugate **1**, and the sodium nitrite solutions were measured at 540 nm. The concentration of the nitrite is calculated by converting absorbance readings to nitrite concentration from standard curve, which is then used to calculate percentage of nitrite released. All experiments were repeated thrice.

Griess assay scheme

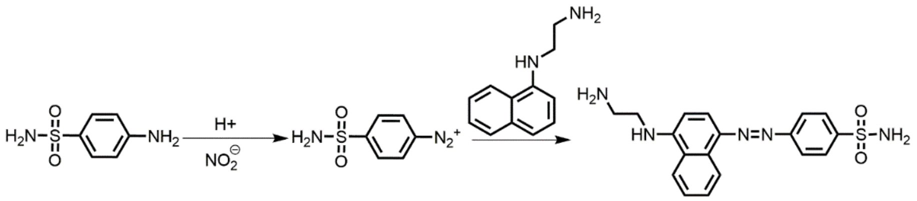

Azo dye (λ_{max} = 540 nm)

Principle of nitrite quantitation using the Griess reaction. Formation of azo dye detectd by absorbance at 540 nm.

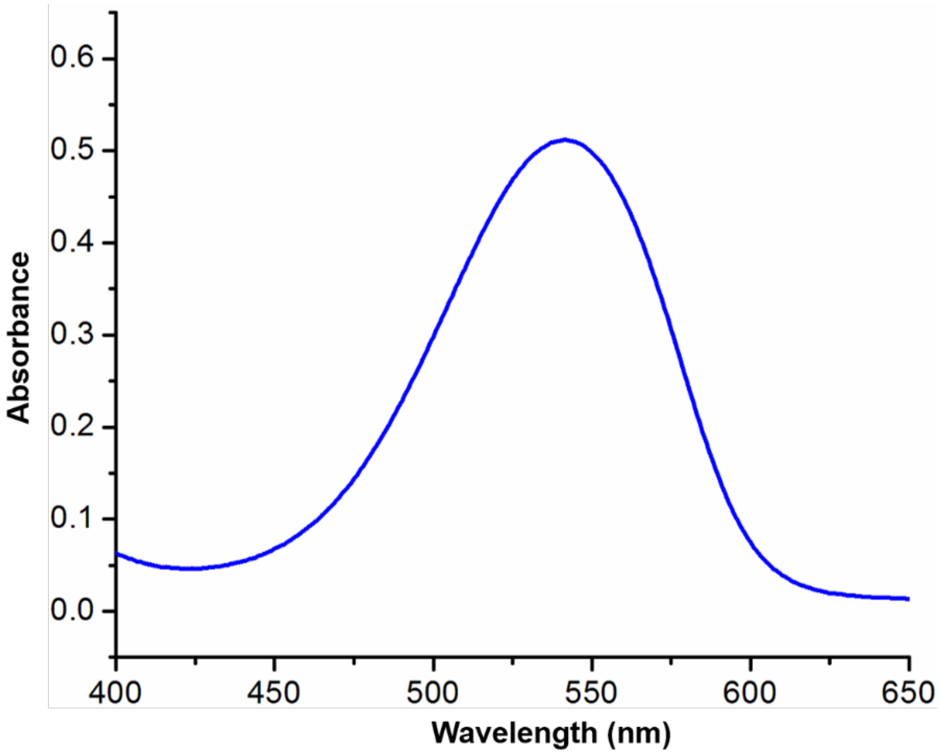

Fig. S3 Representative UV-Vis spectrum of the nitrite containing incubation mixture after incubation of **1** (100 µM**)** with glutathione (5 mM) in PBS at 37 °C followed by treatment with Griess reagent .

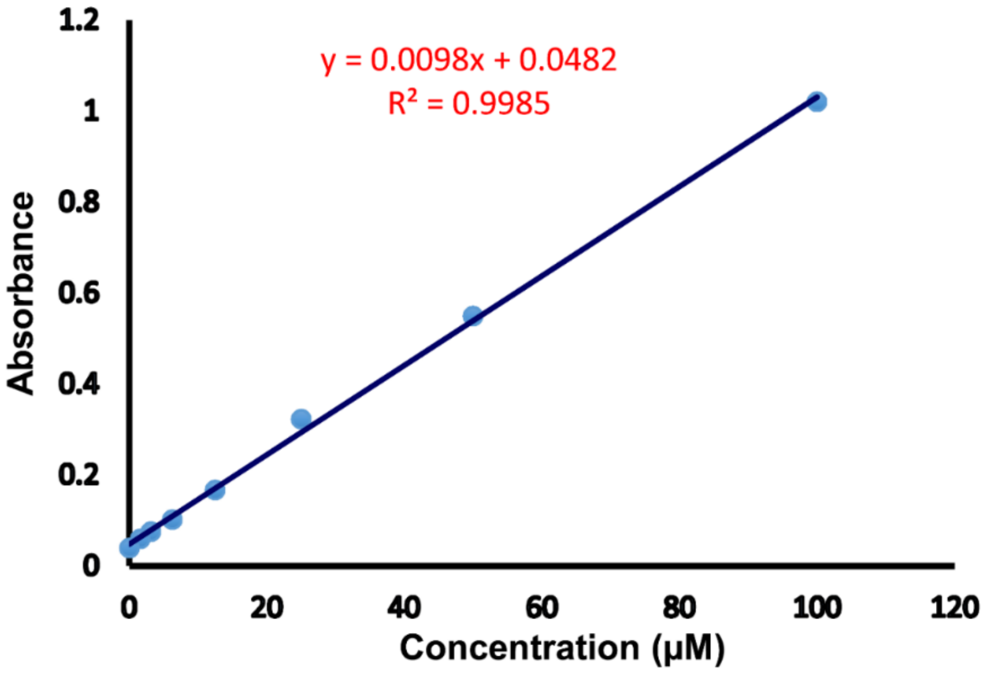

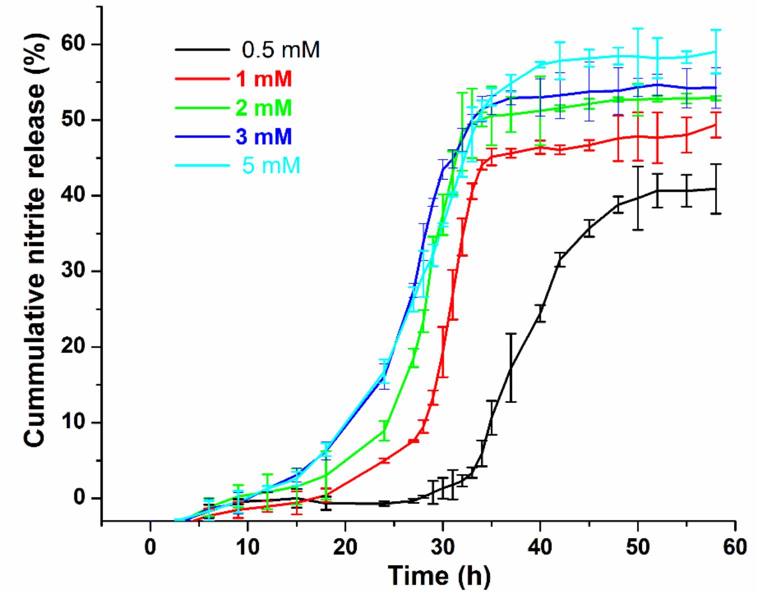

Fig. S4 Standard nitrite (NO_2^-) concentration-absorption curve.

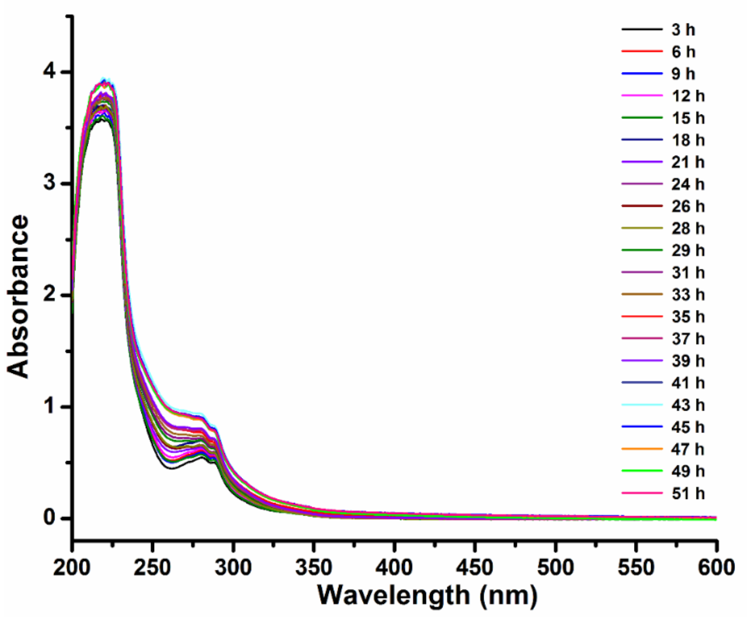

Fig. S5 Nitrite release from **1** as a function of glutathione concentration.

Fig. S6 Time dependent UV Vis spectrum of **1** incubated with glutathione at 37 °C.

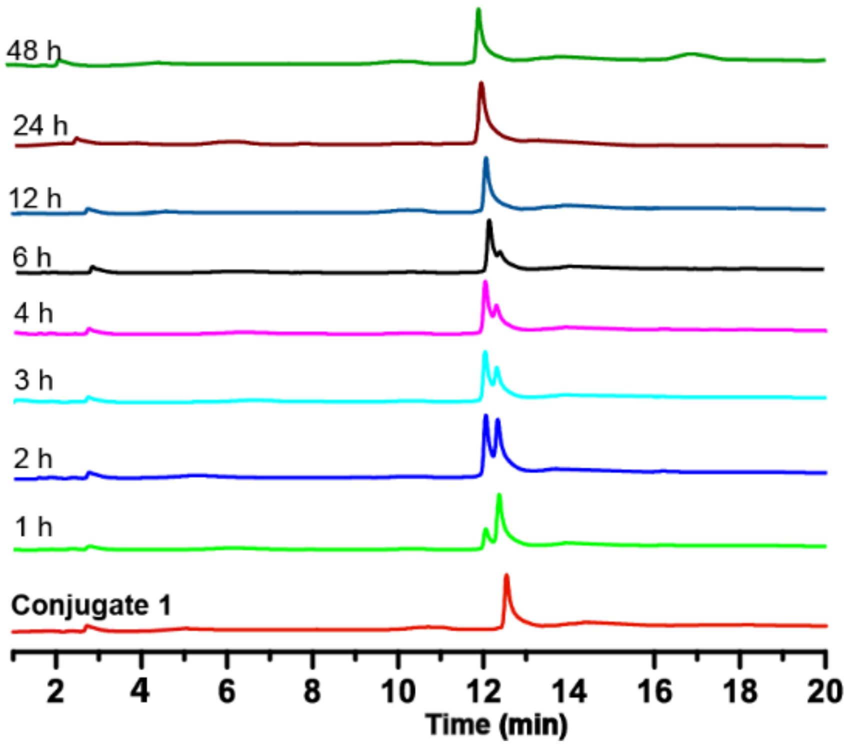

Fig. S7 Time dependent HPLC spectrum of **1** incubated with glutathione in phosphate buffer solution (5X, pH = 7.4) at 37 °C.

Cell culture studies

MTT Assay: MTT assay was performed to check the cytotoxicity of **1** in Neuro2a cells. Cell viability was assessed by measuring formazan produced by the reduction of MTT. Neuro2a cells were incubated in 96-well plate with variable concentrations of **1** such as 25, 50, 100 and 200 μM and incubated for 24 h at 37 °C. The cells in each well were then incubated in culture medium with 10 mg/mL MTT for 4 h at 37 °C. DMSO-methanol was used to solubilize formazan prior to reading using microplate ELISA reader (Thermo; Multiskan™ GO Microplate Spectrophotometer) at 550 nm of wavelength. No cytotoxicity was observed from this assay.

NO release experiment: Flow cytometry was performed using NO indicator DAF-FM diacetate for estimation of NO release inside the Neuro2a cells. Briefly, Neuro2a cells (10^6 cells / mL) were harvested overnight in 6 well plate. These cells were treated with different concentrations of **1** such as 5, 10, 20 and 40 µM and a control compound (nitrated aspirin, 20 µM) and incubated for 24 h in humidified CO_2 incubator at 37 °C. Then cell suspensions were prepared using trypsin and washed thoroughly with serum free DMEM followed by incubation with 5 µM DAF-FM diacetate for 30 min at 37 ^oC. Subsequently, cells were washed with serum free DMEM and incubated for another 30 min in serum free DMEM before analysis in FACS (LSRFORTESA) using FITC filter.

Neurite outgrowth analysis: For analysis of neurite outgrowth in Neuro2a cells, about 5000 cells were seeded in cover glass bottom dish and harvested overnight. The cells were treated with **1** (20 µM) and incubated for 24 h in humidified CO_2 incubator at 37 ^oC. Consequently, cells were analyzed under microscope (Olympus) at DIC mode. Cells with extracellular process extending longer than two cell bodies were considered as neurite-bearing cells. Quantitative evaluation of neurite containing cells and neurite length was carried out using analysis and calculation mode of cellSens software. In neurite outgrowth analysis significant neurite development was observed upon treatment with **1** compared to untreated cells.

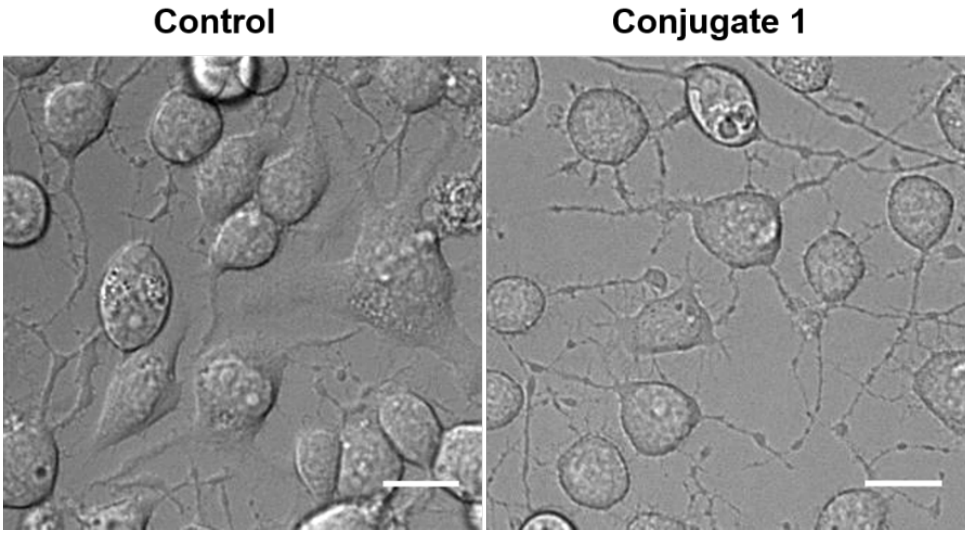

Fig. S8 Zoomed images of neurite out growth in neuron cells (scale bar = 40 µm)

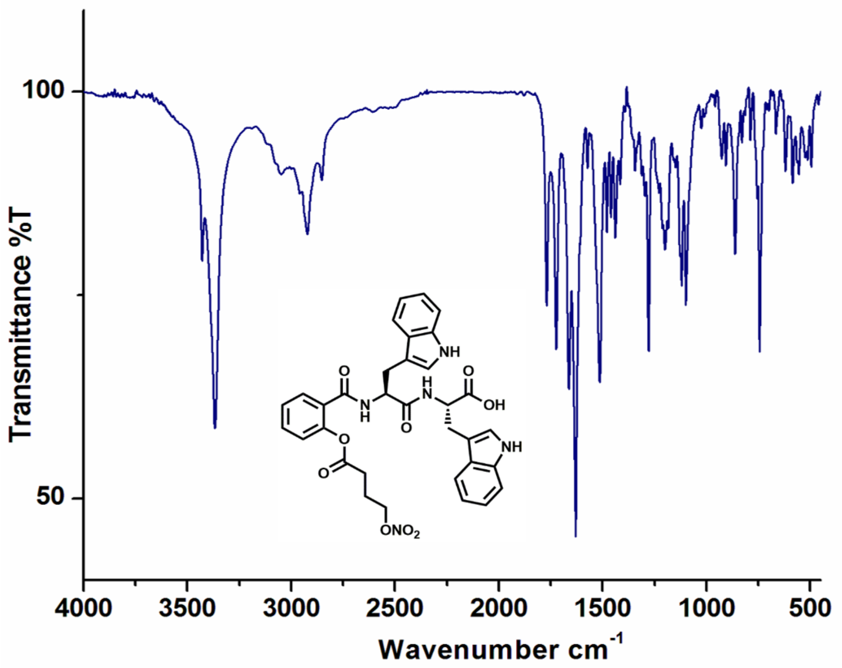

Fig. S9 IR spectrum of **1.**

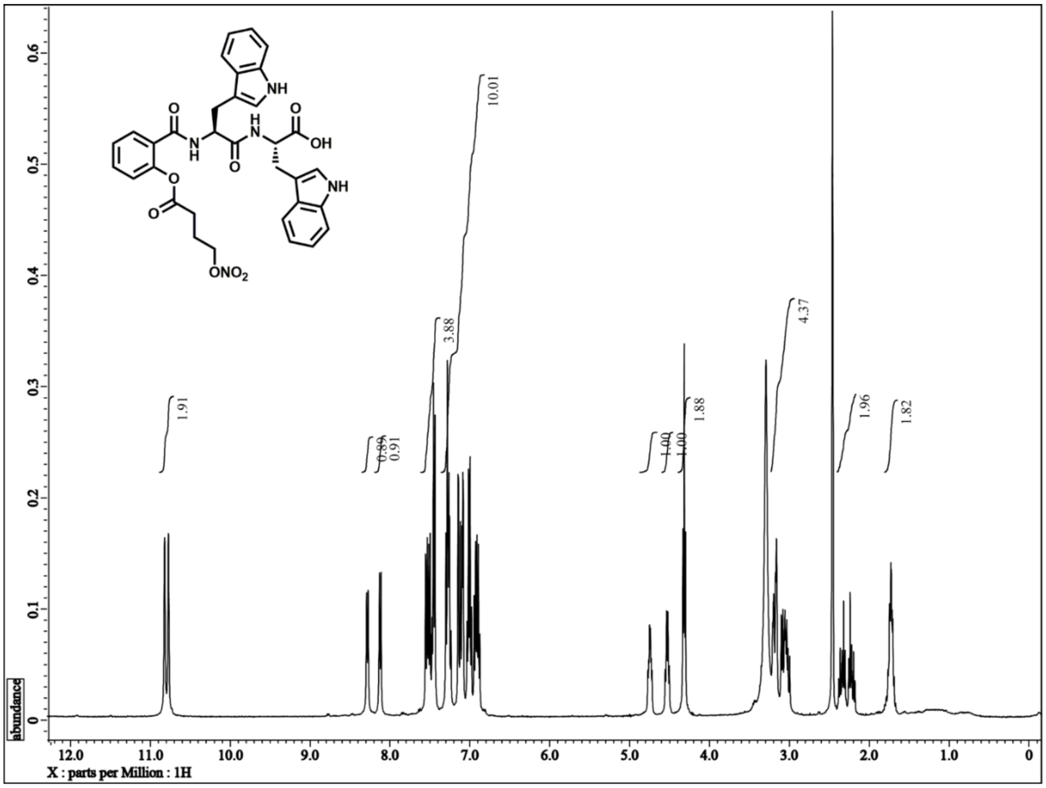

Fig. S10 ^{1}H NMR spectrum of **1.**

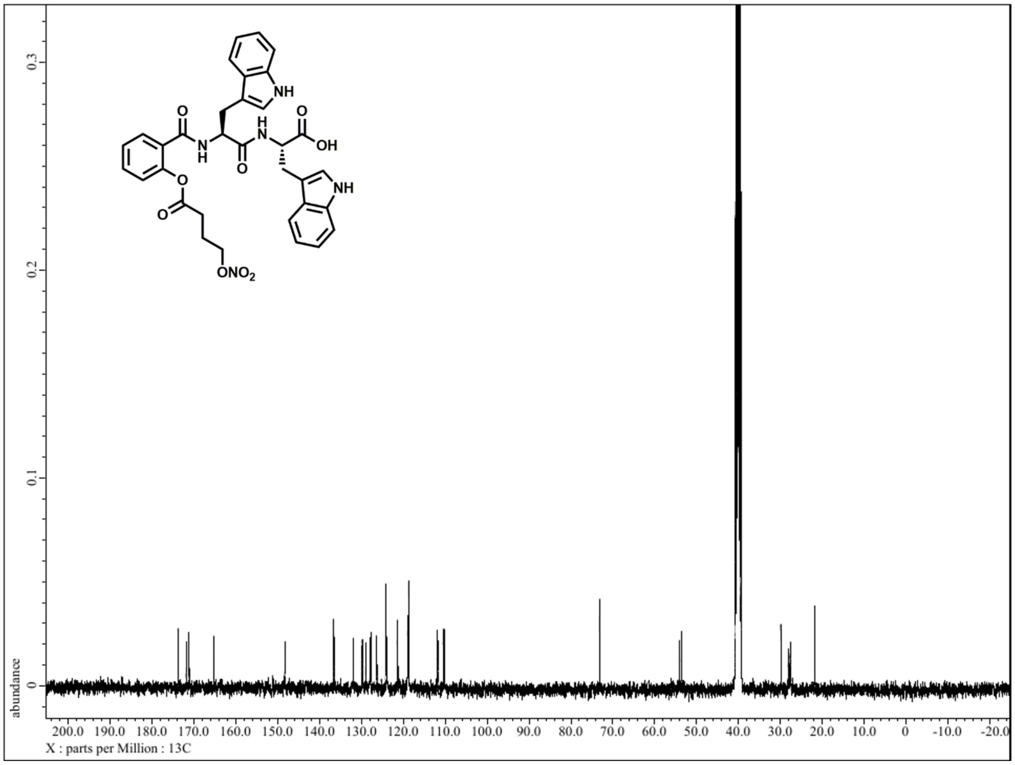

Fig. S11 ^{13}C NMR spectrum of **1**.

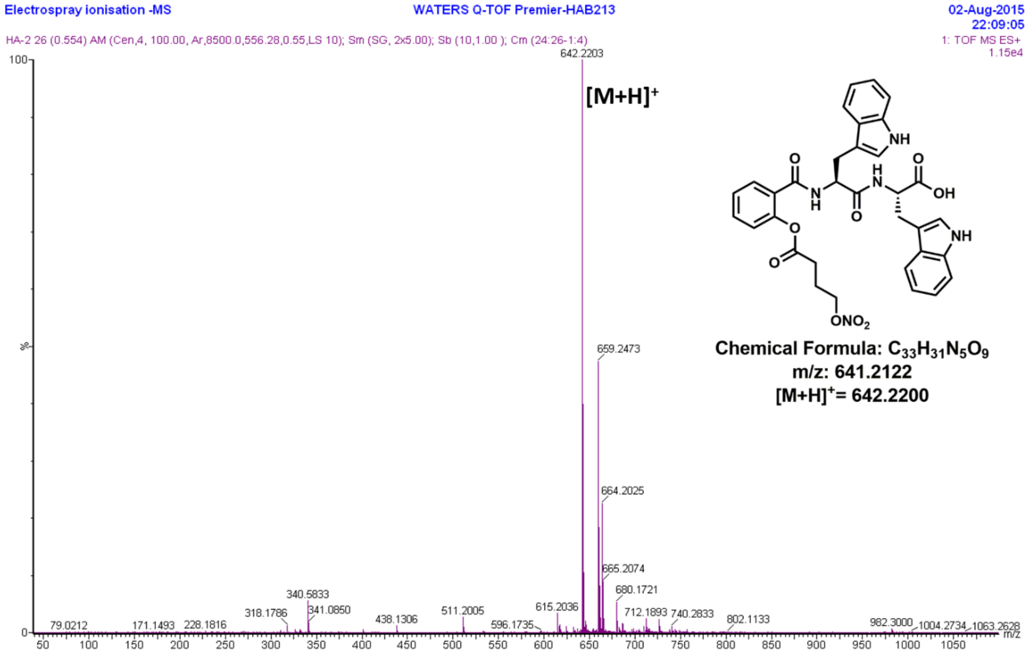

Fig. S12. ESI-HRMS spectrum of **1.**

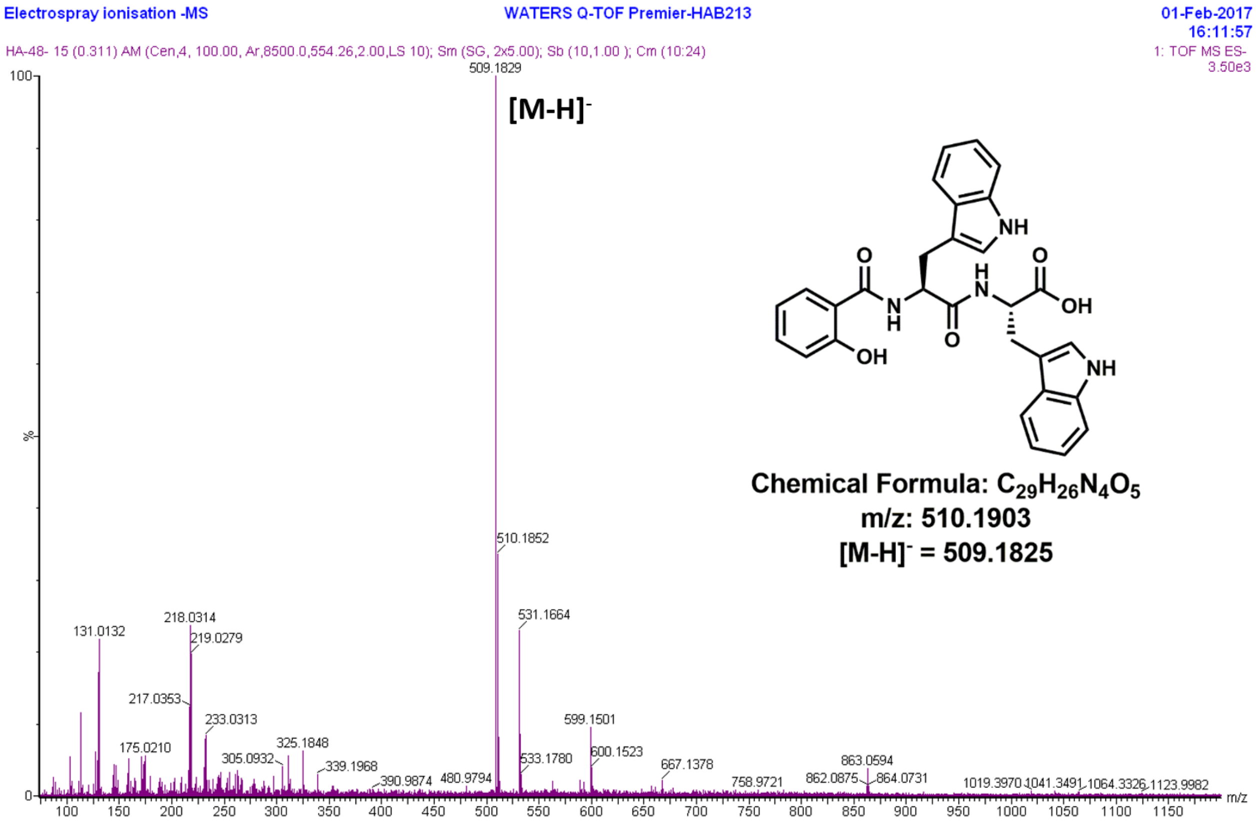

Fig. S13. ESI-HRMS spectrum of **1** after incubation with glutathione for 48 h**.**

References:

L. Fang, M. Wang, S. Gou, X. Liu, H. Zhang, Feng Cao, *J. Med. Chem.* 2014, **57***,* 1116−1120.

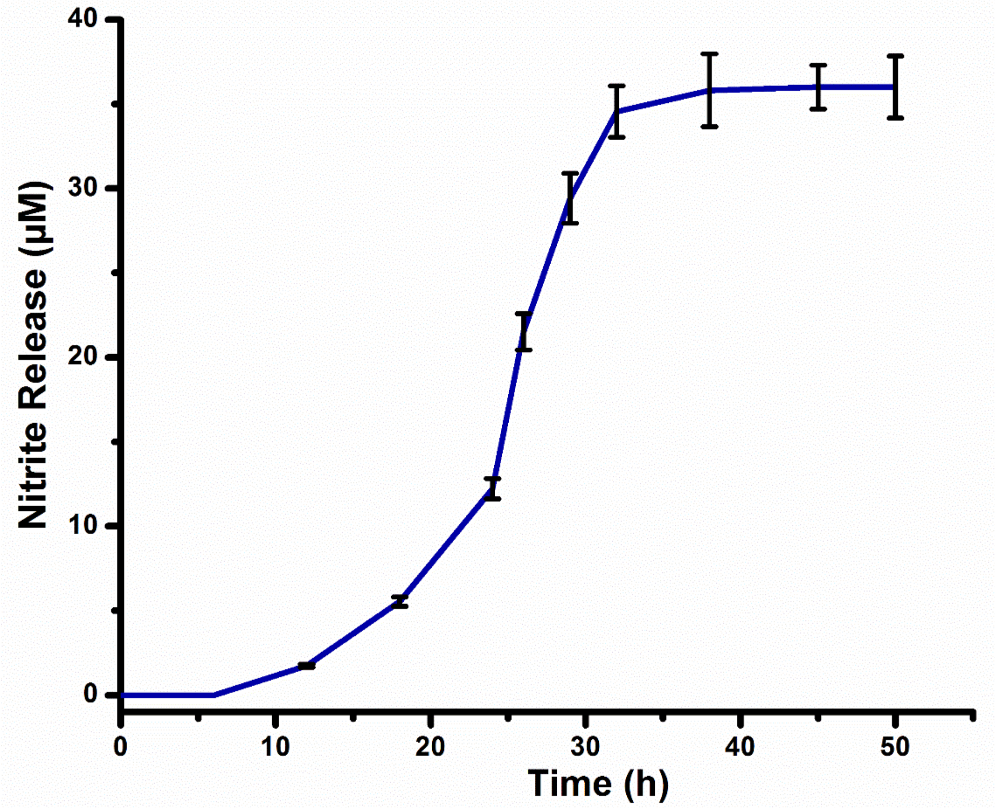

Fig. S14: Nitrite release profile of **1** (100 µM) on incubation with glutathione (5 mM) in phosphate buffer solution (5X, pH 7.4), followed by detection and quantification of nitrite ions by anion exchange chromatography.
